# Supplementary material for: Seminoma and Embryonal Carcinoma Footprints Identified by Analysis of Integrated Genome-Wide Epigenetic and Expression Profiles of Germ Cell Cancer Cell Lines
Source: PLoS One. 2014 Jun 2;9(6):e98330. doi: 10.1371/journal.pone.0098330 (PMC4041891; doi:10.1371/journal.pone.0098330)
Supplement: File S1 — ZIP file containing DMRforPairs output for significant regions. Please start from the html files. (ZIP) [file pone.0098330.s008.zip › figures/1170.pdf]

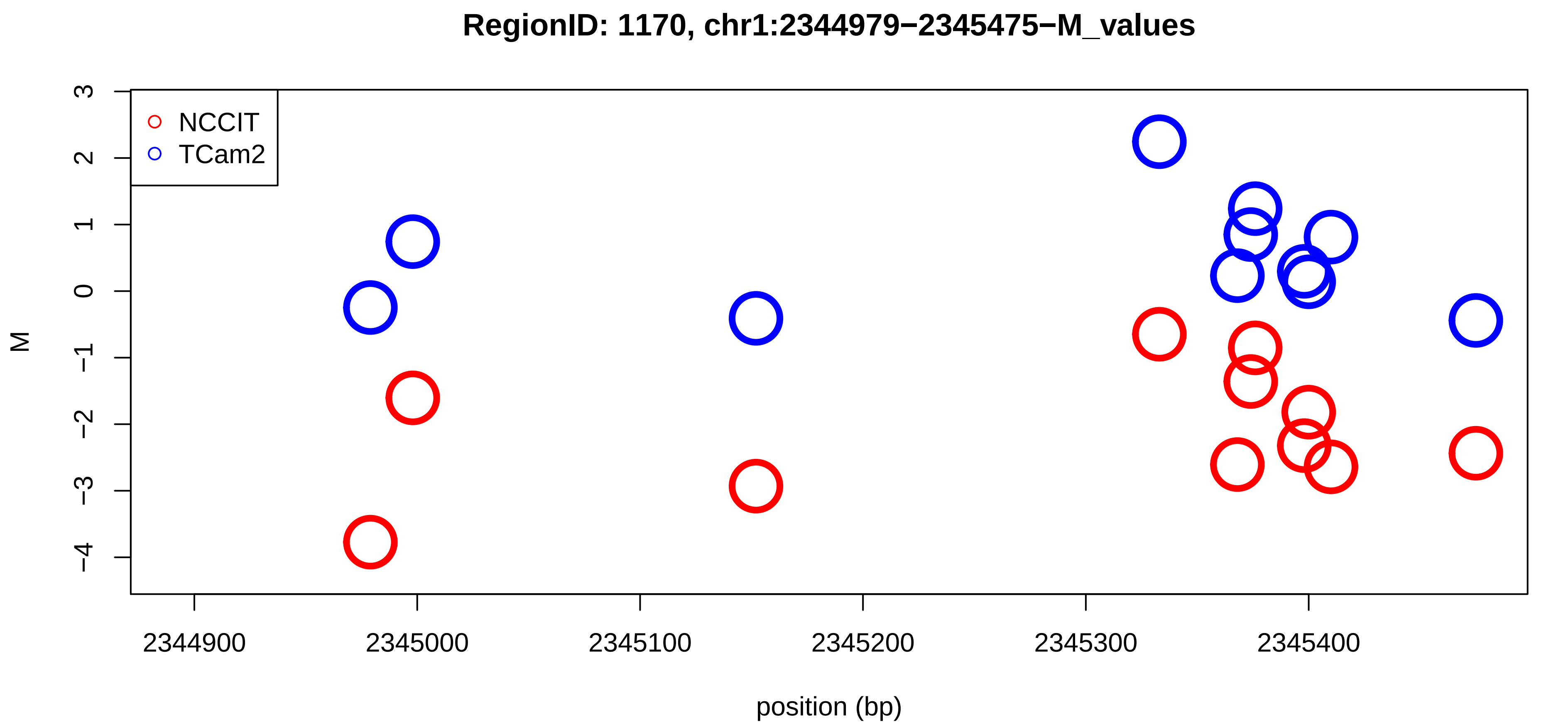
RegionID: 1170, chr1:2344979−2345475−M_values
NCCIT
TCam2
M
3
2
1
0
−1
−2
−3
−4
2344900
2345000
2345100
2345200
2345300
2345400
position (bp)

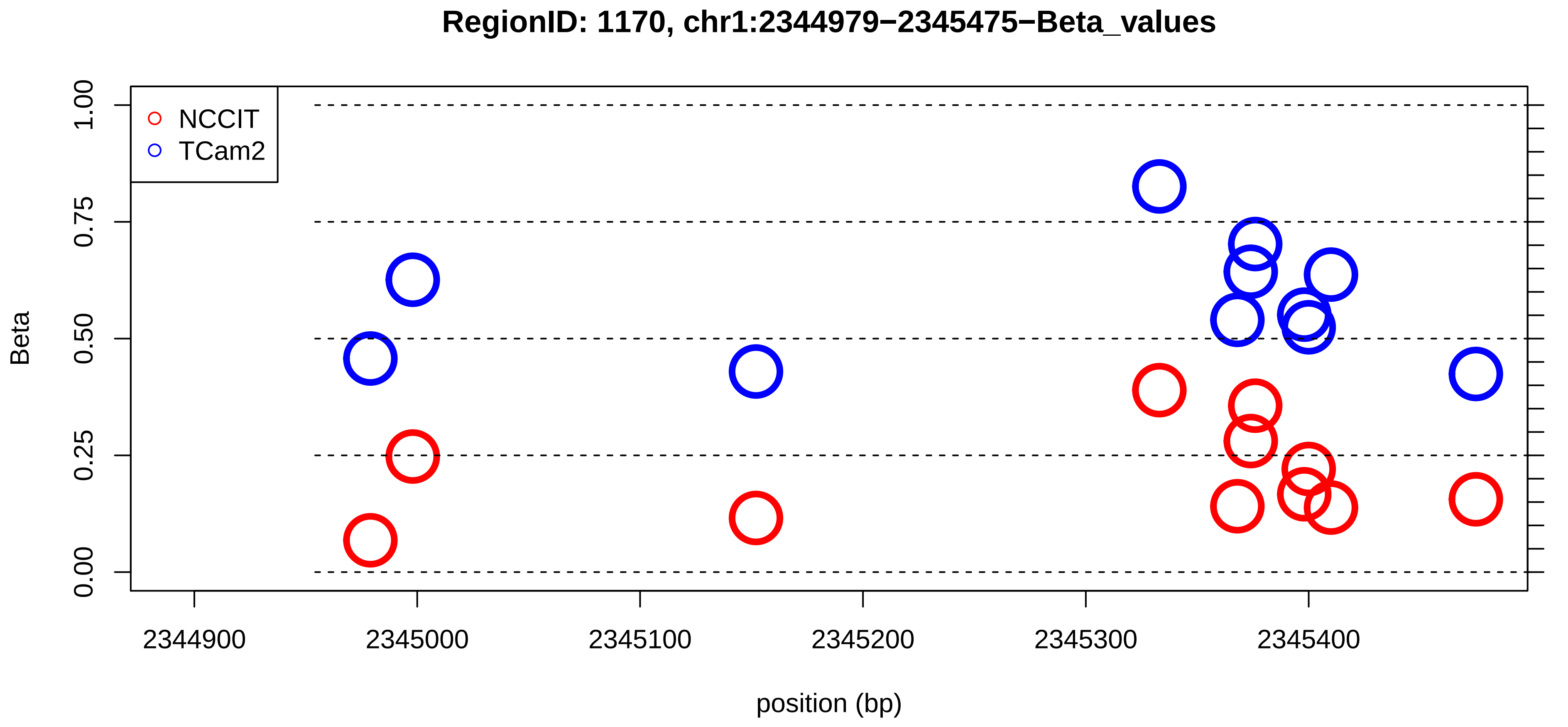
RegionID: 1170, chr1:2344979−2345475−Beta_values
NCCIT
TCam2
Beta
1.00
0.75
0.50
0.25
0.00
2344900
2345000
2345100
2345200
2345300
2345400
position (bp)

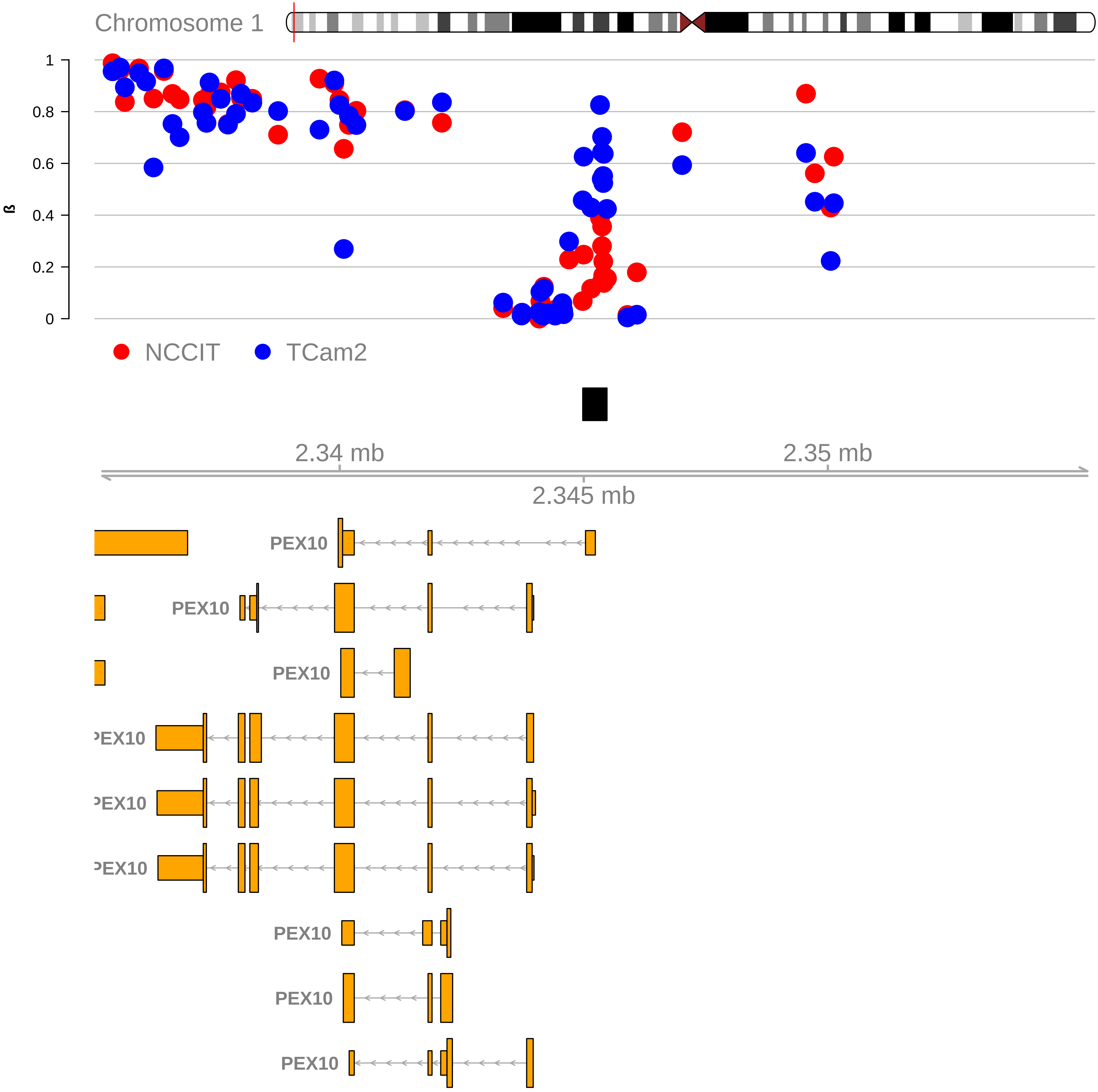
Chromosome 1
1
0.8
0.6
0.4
0.2
0
β
NCCIT
TCam2
2.34 mb
2.35 mb
2.345 mb
PEX10
PEX10
PEX10
PEX10
PEX10
PEX10
PEX10
PEX10
PEX10
